# American Medical Association—The Nashville Meeting

**Published:** 1890-06

**Authors:** 


BUFFALO MEDICAL AND SURGICAL JOURNAL

A MONTHLY REVIEW OF MEDICINE AND SURGERY.

*EDITORS:*

THOS. LOTHROP, M. D. - - - W. W. POTTER, M. D.

All communications, whether of a literary or business character, should be addressed to the editors: 284 FRANKLIN STREET, BUFFALO, N. Y.

### Editorial.

### AMERICAN MEDICAL ASSOCIATION—THE NASHVILLE MEETING.

THE forty-first annual meeting of the American Medical Association, held in Nashville, Tenn., May 20, 21, 22, and 23, 1890, from all points of view must be regarded as one of the most successful medical gatherings that has yet been held under the auspices of this organization.

The Association of Medical Editors, which may be regarded as an auxiliary to the main or parent association, convened at nine o'clock, Monday evening, May 19, 1890, under the presidency of Dr. I. N. Love, of St. Louis, who conducted the meeting with his usual ability. After listening to the papers of Drs. F. L. Sim, of Memphis, and T. D. Crothers, of Hartford, and electing officers for the ensuing year, the association adjourned to partake of the annual banquet. The officers chosen were: President, Dr. F. L. Sim, of Memphis; Vice-President, Dr. Frank Woodbury, of Philadelphia; Secretary and Treasurer, Dr. J. C. Culbertson, of Cincinnati.

The Association met in general session at the Vendome Theater, Tuesday, at 11 o'clock A. M., when the President, Dr. E. M. Moore, of Rochester, delivered his annual address in which he dealt largely with the problems of National and State Hygiene. It was an able discourse, as might be expected from so learned an author, and will well repay careful reading by every physician.

At 3 o'clock the several sections convened in their appointed places of meeting, which were all conveniently located and well adapted to their purposes. During the three days following, over two hundred papers were read and discussed—an enormous amount of work to dispose of, when it is remembered that the sections, ten in number, hold their sessions in the afternoons only, the mornings being devoted to the general meetings. At the latter the business and legislative affairs are disposed of, and the general addresses on the Practice ot Medicine, Surgery, and State Medicine delivered.

Dr. W. T. Briggs, of Nashville, was elected president for the ensuing year, and Washington chosen as the place of meeting, the date being the first Tuesday in May, 1891.

The exhibits of instruments, appliances, surgical tables, pharmaceutical preparations, and foods, at Amusement Hall, were elaborate and interesting. These exhibits are far more instructive than one would suppose who had never visited them, and are by no means to be regarded from merely a trade standpoint. The pharmacist and chemist, the instrument maker, and the manufacturers of the various tables and ingenious appliances, all mark the progress making in medicine and surgery quite as plainly as do the scientific papers read at the section meetings. The exhibitors are not to be regarded as mere distributers of samples, but are, for the most part, men of education, and skilful in their several departments. The address of Mr. Smith, on Friday, in presenting the handsome easy chair to Dr. Lindsley, of Nashville, in behalf of the exhibitors, may be cited in support of the foregoing statement; it was eloquent, scholarly, finished and appropriate, and it is difficult to see how it could have been improved upon by any statesman in the land.

The medical colleges, through their representatives, met on Wednesday and effected an organization looking to the advancement of educational standards, but no opinion can now be ventured as to the good that will be accomplished thereby.

Finally, though not of the last importance, we must speak of the social side of the Nashville annual, for no account of this great occasion would be complete that omitted to do justice to this part of the meeting. While in this beautiful city of the mid-south, so noted for its educational institutions, handsome women, and attractive scenery, it was to be expected that much would be done for the entertainment of the guests, no conception could be formed in advance that would approach the realities of what this hospitable people were capable of, or of the extent of their generosity in the direction named. While it is impossible to particularize with the space at our command, we may say that from the moment the visitors arrived on Monday, until the last one departed on Saturday, there was one continual round of social entertainment that was of the most delightful sort. Receptions, excursions, dinners, teas, and visits; rides, drives, and walks were all happily appointed, and reflect the greatest credit upon the citizens of Nashville, who seemed to vie with each other in devoting their time and money to the entertainment of the American Medical Association.

The ladies' reception at the Maxwell House, on Wednesday afternoon, is to be specially mentioned as a novel feature, which brought the visitors and the Nashville ladies to a delightful acquaintanceship that could not have been accomplished in any other way; and to the good taste and judgment of Mrs. S. A. Champion, in carrying out this part of the programme, all praise must be accorded. We are as loth to leave this interesting subject as we were pained to bid adieu to Nashville and its charming people; and we close, by saying, as we began, that the meeting must be pronounced a success, both from a scientific and social standpoint.

To indicate the degree of perfection of the organization on entertainments we subjoin the list of the

LADIES' RECEPTION COMMITTEE.

Mrs. J. B. Lindsley, chairman.

(*a*) *Section on Maxwell House.* Mrs. S. A. Champion, chairman; Mesdames Thos. Plater, W. H. Payne, W. H. Peck, C. A. Miller, Van Kirkman, Leslie Warner, Mat Williams, Percy Warner, J. H. Eakin, R. F. Weakley, W. S. Bransford, J. M. Harding, W. W. Berry; Misses Margerie Spurr, Annie Gray, White May, Dovie Briggs, Henrietta Kendrick, May Belle Keith, Mary Reese, Louise Lindsley, Anna Brennan, Emma Morrow.

(*b.*) *Section on "The Duncan."* Mrs. J. M. Head, chairman. Mesdames M. L. Hicks, H. H. Lurton, Dr. Paul F. Eve, Nathaniel Baxter, Sr., J. F. Wheless, Myron Peck, Dr. J. H. Blanks, Albert D. Marks, Wm. M. Duncan; Misses Mary McGavock, Marshall, Mamie Vaughn, Roberta Jones, Mamie Duncan, Annie D. Lindsley.

(*c.*) *Section on Baxter Café and Nicholson House.* Mrs. M. P. McGuire, chairman. Mesdames Chas. Thurman, Isaac Reese, Jere Baxter, Dr. N. D. Richardson, J. P. W. Brown, Chas. H. Eastman, John Thomas, Jr., John Childress, J. C. Warner; Misses Mamie Rogers, Lillie Morrow, Annie Porterfield.

(*d.*) *Section on other Hotels.* Mrs. W. D. Haggard, chairman. Mesdames Ed. Buford, Dr. Richard Douglas, Dr. Duncan Eve, Mark Cockrill, M. P. House, D. R. Dorris, Robert Riddle; Misses Mattie Nelson, Birdie Brown, Mary Bright, Mattie Spurr.

(*e.*) *Section on Boarding Houses and Homes.* Mrs. M. B. Pilcher, chairman. Mrs. Mary Clare; Misses Gillespie, Gretta Williams, Sallie Foard, Annie M. Callender, Lizzie Atchison, Madora Jones.

